# Data of MSCs combined with LITUS treatment to improve cognitive impairment in a moderate traumatic brain injury model in rats

**DOI:** 10.1016/j.dib.2023.108947

**Published:** 2023-02-01

**Authors:** Xinyu Yao, Wenzhu Wang, Yue Li, Zhendong Cao, Yongheng Wang, Yi Yuan, Xiaoling Li, Xin Liang, Lanxiang Liu, Yan Yu

**Affiliations:** a Graduate School of Chengde Medical University, Hebei Province, China; b First Hospital of Qinhuangdao, Hebei Province, China; c Beijing Key Laboratory of Neural Injury and Rehabilitation, China Rehabilitation Research Center, Beijing, China; d Guizhou University of Traditional Chinese Medicine, Guizhou Province, China; e Department of Neurosurgery, First Hospital of Qinhuangdao, Hebei Province, China; f School of Electrical Engineering, Yanshan University, Hebei Province, China; g Applying Chemistry Key Lab, Yanshan University, Hebei Province, China

**Keywords:** Traumatic brain injury, Mesenchymal stem cells, Low-intensity transcranial ultrasound, Cognitive dysfunction, Combined therapy

## Abstract

Here, we treated moderately traumatic brain injury (TBI) rats with different modalities, including transplantation with mesenchymal stem cells (MSCs), treatment with low-intensity transcranial ultrasound stimulation (LITUS), and a combination of the two. After the TBI rat model was established, MSCs (*in situ* injection within 24 h after injury), LITUS (continuous uninterrupted treatment for 28 days) or combined MSCs + LITUS were administered, and mNSS score, performance of behavior and multiple protein levels were compared between groups by behavioral observation, neurological function assessment and pathological analysis. Nestin, neuron-specific enolase (NSE), growth-associated protein 43 (GAP-43) and postsynaptic density protein (PSD-95) were significantly increased and glial fibrillary acidic protein (GFAP) was significantly decreased in the hippocampus of rats in the combination treatment group; brain-derived neurotrophic factor (BDNF), tumor necrosis factor-α (TNF-α) and aquaporin-4 (AQP-4) were significantly decreased in the injured peripheral cortex. The result of mNSS scores was: TBI group > LITUS group > MSCs group > MSCs+LITUS group > sham group. The alternate correct rate of Y-maze was: sham group > MSCs+LITUS group > MSCs group > LITUS group > TBI group. This data compares the efficacy of MSCs, LITUS, and combination therapy on the level expression of stem cell differentiation related proteins, synaptic plasticity-related proteins, neurotrophic factors, inflammatory factors, and edema-related proteins after TBI by quantitative pathological examination. For a complete description, interpretation, and discussion of the data refer to the article in press [1].

**Specifications Table**

**Table:** 

Subject	Biology
Specific subject area	Cognitive neuroscience and neural regeneration
Type of data	Tables, figures
How data were acquired	Rat neurological function scores were rated double-blind by two investigators according to the mNSS scoring criteria. The spontaneous alternation rate of the rats was calculated by the formula: correct alternation rate = number of correct alternations/(total number of arm entries - 2) × 100%. The expression levels of relevant proteins and related genes in rat brain tissue were detected by immunohistochemistry, western blot and RT–PCR.
Data format	Raw, analyzed
Parameters for data collection	MSC injection (within 24 h after TBI), LITUS treatment (continuous and uninterrupted for 28 days), and combined treatment (MSC injection within 24 h after TBI and continuous uninterrupted 28 day LITUS treatment) were performed on different groups of rats after TBI. Treatment effects were compared between the different intervention and control groups.
Data source location	China Rehabilitation Research Center, Beijing Key Laboratory of Neural Injury and Rehabilitation, Beijing, China
Data accessibility	The data is stored in the Mendeley database. DOI:10.17632/h4jmbnkmjc.1 https://data.mendeley.com/datasets/h4jmbnkmjc
Related research article	Yao X, Wang W, Li Y, Cao Z, Wang Y, Yuan Y, Li X, Liang X, Yu Y, Liu L. Study of the mechanism by which MSCs combined with LITUS treatment improve cognitive dysfunction caused by traumatic brain injury. Neurosci Lett. 2022 Aug 4;787:136,825. DOI:10.1016/j.neulet.2022.136825.

## Value of the Data

• These data are useful because there is no effective treatment for TBI, which has high morbidity, mortality, and disability rates, and these data provide a reference for the treatment of TBI.
• Clinicians and researchers in neurosurgery and rehabilitation, as well as the public, will benefit from this research, as the results have important implications for human health and disease.
• These data can provide a reference for the treatment and rehabilitation after clinical TBI to improve the quality of life and survival of TBI patients. These data also
• These data indicate the need for more research on stem cell combination therapy for TBI and encourage future studies to find more appropriate treatment parameters

## 1 Data Description

We applied MSCs, LITUS or MSCs+LITUS to rats with TBI and compared them with the control group without treatment as well as the sham group. The mNSS values of rats in each group on days 1, 3, 7, 14, 21, and 28 are shown in Table 1, and the Y-maze spontaneous alternation rate is shown in Table 2. Nesin, NSE, and GFAP protein expression in the DG area of the hippocampus; GAP-43 and PSD-95 protein and mRNA expression in the CA1 area; and BDNF, TNF-α, and AQP4 protein and mRNA expression values in the injured perirhinal cortex were detected by immunohistochemistry, western blot and RT–PCR in each group of rats, as shown in Table 3, Table 4, Table 5, Table 6.

**Table 1:** The mNSS values in each group of rats at 1, 3, 7, 14, 21 and 28 days after TBI (n=9, mean±SD).

	1d	3d	7d	14d	21d	28d
Sham	0	0	0	0	0	0
TBI	11.889±1.054	11.111±1.269	9.222±0.972	7.111±1.167	6.889±0.928	6.556±0.726
TBI+MSCs	11.778±1.394	10.889±1.054	8.444±0.726	5.556±0.527^⁎⁎^	5.222±0.667^⁎⁎⁎^	4.556±0.527^⁎⁎⁎^
TBI+LITUS	11.111±1.269	10.111±1.054	8.889±0.928	5.778±0.833^⁎⁎^	5.444±0.527^⁎⁎^	4.778±0.667^⁎⁎⁎^
TBI+MSCs+LITUS	10.889±1.167	9.778±1.092*	7.778±0.972^⁎⁎^	4.556±1.014^⁎⁎⁎^	4.222±1.302^⁎⁎⁎^	3.222±0.972^⁎⁎⁎^

Data are presented as the mean±SD. Values are compared with the TBI group. Differences were analyzed by one-way analysis of variance.

*p < 0.05.

^⁎⁎^p < 0.01.

^⁎⁎⁎^p < 0.001.

**Table 2:** The spontaneous alternation rate on the Y-maze for each group of rats 28 days after TBI (n=6, mean±SD).

	Spontaneous alternation rate
Sham	0.860±0.046^⁎⁎⁎^
TBI	0.469±0.085
TBI+MSCs	0.651±0.064^⁎⁎⁎^
TBI+LITUS	0.597±0.026^⁎⁎^
TBI+MSCs+LITUS	0.777±0.045^⁎⁎⁎^

Data are presented as the mean ± SD. Values are compared with the TBI group. Differences were analyzed by one-way analysis of variance.

^⁎⁎^p < 0.01.

^⁎⁎⁎^p < 0.001.

**Table 3:** Mean optical density (AOD) values of Nestin, NSE, and GFAP in the hippocampal DG area of each group of rats 28 days after TBI (n=6, mean ± SD).

	Nestin	NSE	GFAP
Sham	0.011±0.002	0.126±0.010	0.150±0.024^⁎⁎⁎^
TBI	0.019±0.005	0.132±0.008	0.232±0.015
TBI+MSCs	0.169±0.013^⁎⁎⁎^	0.149±0.013*	0.193±0.014^⁎⁎^
TBI+LITUS	0.021±0.003	0.139±0.007	0.219±0.023
TBI+MSCs+LITUS	0.206±0.022^⁎⁎⁎^	0.167±0.017^⁎⁎⁎^	0.171±0.014^⁎⁎⁎^

Data are presented as the mean ± SD. Values are compared with the TBI group. Differences were analyzed by one-way analysis of variance.

*p < 0.05.

^⁎⁎^p < 0.01.

^⁎⁎⁎^p < 0.001.

**Table 4:** The mean optical density (AOD) values of GAP-43 and PSD95 in the hippocampal CA1 region of each group of rats 28 days after TBI (n=6, mean±SD).

	GAP-43	PSD-95	BDNF
Sham	0.359±0.030^⁎⁎⁎^	0.287±0.023^⁎⁎⁎^	0.219±0.016
TBI	0.217±0.016	0.166±0.024	0.240±0.022
TBI+MSCs	0.265±0.029^⁎⁎^	0.219±0.018^⁎⁎⁎^	0.295±0.023^⁎⁎⁎^
TBI+LITUS	0.248±0.030*	0.203±0.026^⁎⁎^	0.268±0.016*
TBI+MSCs+LITUS	0.300±0.019^⁎⁎⁎^	0.251±0.018^⁎⁎⁎^	0.350±0.030^⁎⁎⁎^

Data are presented as the mean ± SD. Values are compared with the TBI group. Differences were analyzed by one-way analysis of variance.

*p < 0.05.

^⁎⁎^p < 0.01.

^⁎⁎⁎^p < 0.001.

**Table 5:** Relative protein and mRNA expression levels of GAP-43 and PSD95 in the hippocampal CA1 region of each group of rats 28 days after TBI (n=6, mean±SD).

	GAP-43		PSD-95	
	Ratio of Densitometric Unit	mRNA	Ratio of Densitometric Unit	mRNA
Sham	0.632±0.038^⁎⁎⁎^	1.027±0.157^⁎⁎⁎^	0.704±0.025^⁎⁎⁎^	1.053±0.190^⁎⁎^
TBI	0.401±0.053	0.432±0.079	0.430±0.049	0.458±0.047
TBI+MSCs	0.501±0.034^⁎⁎⁎^	0.604±0.046^⁎⁎^	0.539±0.048^⁎⁎⁎^	0.607±0.039^⁎⁎^
TBI+LITUS	0.452±0.034*	0.566±0.088*	0.520±0.061^⁎⁎^	0.570±0.039*
TBI+MSCs+LITUS	0.579±0.040^⁎⁎⁎^	0.773±0.055^⁎⁎⁎^	0.612±0.034^⁎⁎⁎^	0.736±0.059^⁎⁎⁎^

Data are presented as the mean ± SD. Values are compared with the TBI group. Differences were analyzed by one-way analysis of variance.

*p < 0.05.

^⁎⁎^p < 0.01.

^⁎⁎⁎^p < 0.001.

**Table 6:** Relative protein and mRNA expression levels of BDNF, TNF-α and AQP4 in the injured perirhinal cortex of rats in each group 28 days after TBI (n=6, mean ± SD).

	BDNF		TNF-α		AQP4	
	Ratio of Densitometric Unit	mRNA	Ratio of Densitometric Unit	mRNA	Ratio of Densitometric Unit	mRNA
Sham	0.414±0.026	1.196±0.361	0.203±0.032^⁎⁎⁎^	1.202±0.211^⁎⁎⁎^	0.141±0.028^⁎⁎⁎^	1.165±0.151^⁎⁎⁎^
TBI	0.461±0.037	1.390±0.189	0.410±0.022	2.353±0.228	0.321±0.038	2.130±0.152
TBI+MSCs	0.549±0.054^⁎⁎^	1.759±0.117*	0.330±0.027^⁎⁎⁎^	1.929±0.155*	0.267±0.043*	1.901±0.093^⁎⁎^
TBI+LITUS	0.515±0.047*	1.779±0.108*	0.314±0.011^⁎⁎⁎^	1.858±0.117*	0.249±0.039^⁎⁎^	1.828±0.076^⁎⁎⁎^
TBI+MSCs+LITUS	0.625±0.047^⁎⁎⁎^	2.127±0.194^⁎⁎^	0.262±0.034^⁎⁎⁎^	1.651±0.061^⁎⁎^	0.204±0.030^⁎⁎⁎^	1.682±0.119^⁎⁎⁎^

Data are presented as the mean ± SD. Values are compared with the TBI group. Differences were analyzed by one-way analysis of variance.

*p < 0.05.

^⁎⁎^p < 0.01.

^⁎⁎⁎^p < 0.001.

## 2 Experimental Design, Materials and Methods

### 2.1 Rats and Groups

Ninety rats were randomly divided into five groups: ①control group; ②TBI group; ③TBI+MSCs group; ④TBI+LITUS group; ⑤TBI+MSCs+LITUS group. A rat TBI model was established by controlled cortical impact (CCI) method using a pneumatic cranial precision percussion instrument (68099II, RWD, Shenzhen, Guangdong, China) [2] with the following relevant parameters: according to the rat brain stereotaxic map, a 5 mm diameter bone flap was grinding and drilling in the right parietal lobe (2.5 mm right of the sagittal line and 3.8 mm posterior to the coronal line of fontanelle), and the impact parameters were set as follows: the impact head diameter was 5 mm, the impact velocity was 5 m/s, the depth was 2.5 mm subdural, and the duration of impact was 1 s. MSCs were transplanted *in situ* at the central site of injury within 24 h after injury, and the relevant parameters were as follows: the cell suspension concentration was 2.5 × 10^7^ cells/ml, the injection volume was 2 µL, the depth was 2.5 mm, the speed was 1 µL/min, and the needle retention time was 5 min [3]. LITUS treatment was performed daily without interruption for 28 days using an ultrasound stimulator (DK-102T, Dukang, Hebei, Shijiazhuang, China) with the following parameters: ultrasound frequency of 800 kHz, pulse duration of 10 ms, and pulse repetition period of 60 ms. The Isppa value was 1.2 W/cm^2^.

### 2.2 Behavioral Tests

Nine rats were randomly selected from each group, and mNSS scores were performed on days 1, 3, 7, 14, 21, and 28 after modeling [4]: the range of scores was 0–18, with higher scores representing more severe craniocerebral injury. Six rats were randomly selected from each group and the Y-maze test was performed on the 28th day after modeling: the Y-maze consisted of three 30 × 8 × 15 cm arms placed at a 120° angle. Rats were recorded for 8 min after 2 min of free exploration in the maze, and entering three different arms consecutively was considered as one correct alternation. The alternation index is calculated as: correct alternation rate = number of correct alternations/(total number of arm feeds - 2) × 100%.

### 2.3 HE Staining and Immunohistochemistry

**Fig. 1:**
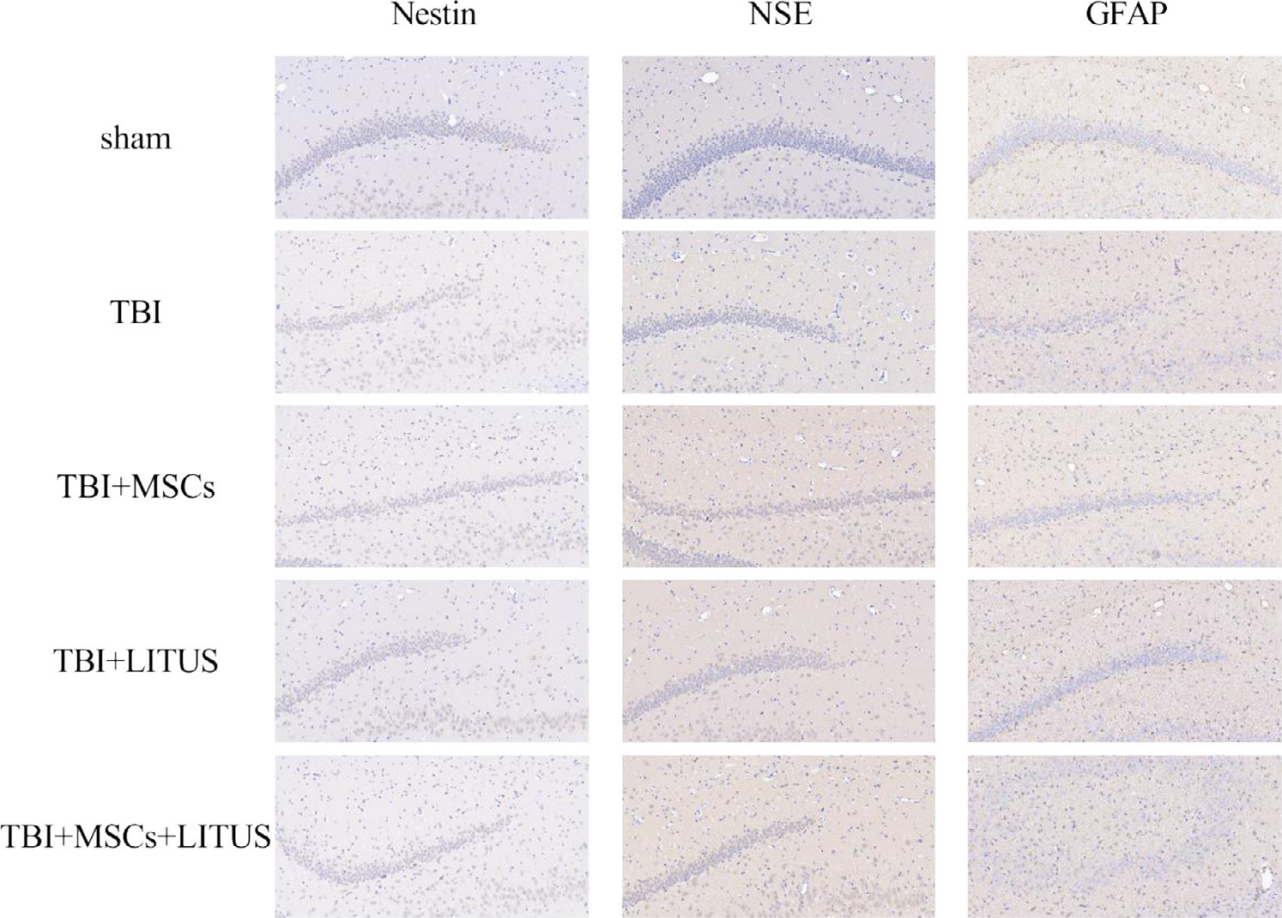
Immunohistochemical staining of the DG region of the hippocampus.

**Fig. 2:**
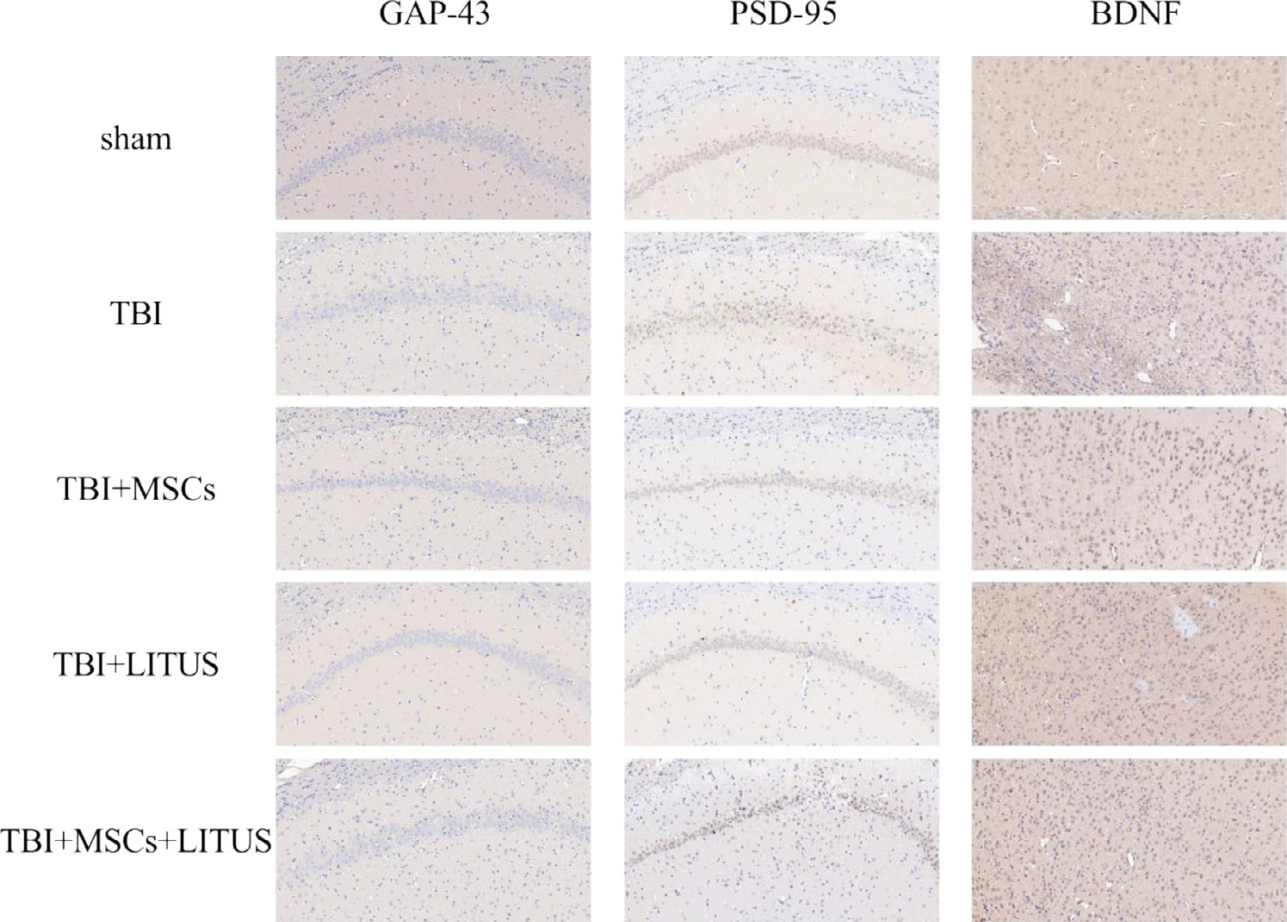
Immunohistochemical staining of hippocampal CA1 region and damaged peripheral cortex.

After 28 days of modeling, six rats were randomly selected from each group, anesthetized by intraperitoneal injection of 2% sodium pentobarbital (2 ml/kg), and 4% paraformaldehyde was perfused to extract the brain for HE staining and immunohistochemical staining, as in the previous experiments [5]. The antibodies used for immunohistochemistry were as follows: rabbit monoclonal anti-Nestin (1:200; ab105389, abcam, UK), rabbit monoclonal anti-NSE (1:200; ab78757, abcam, UK), rabbit monoclonal anti-GFAP (1:200; ab7260, abcam, UK), rabbit monoclonal anti-GAP-43 (1:200; ab75810, abcam, UK), rabbit polyclonal anti-PSD-95 (1:200; ab18258, abcam, UK), and rabbit monoclonal anti-BDNF (1:200; ab108319, abcam, UK). Images were taken with an Olympus BX43F light microscope and the average optical density (AOD) of the immunoreactive signal was evaluated using Image-Pro-Plus 5.1 software (Media Cybernetics, Silver Spring, MD, USA): AOD = IOD/average area (IOD/average area = sum of IOD/area). Figs. 1 and 2

### 2.4 Western Blotting

The remaining rats were sacrificed and the brains were removed, the hippocampal tissue and the peri‑injured cortex were isolated, and 100 mg samples were taken to detect the amount of protein by immunoblotting [6]. The antibodies used were as follows: rabbit monoclonal anti-GAP-43 (1:1000; ab75810, Abcam, UK), rabbit monoclonal anti-PSD-95 (1:1000; ab18258, Abcam, UK), rabbit monoclonal anti-BDNF (1:1000; ab108319, Abcam, UK), rabbit monoclonal anti-TNF-α (1:1000; ab183218, Abcam, UK), rabbit monoclonal anti-AQP-4 (1:1000; ab128906, Abcam, UK), and rat polyclonal anti-β-actin (1:1000; 4970S, Cell Signaling Technology, USA). Bound antibodies were detected using an ECL western blotting detection system kit (abs920, Absin Biotechnology Co., Ltd., Shanghai, China) and exposed using a ChemiDOC™ XRS+ system with Image Lab™ software (Bio-Rad Life Medical Products Co., Ltd., Shanghai, China).

### 2.5 Semiquantitative Reverse Transcription-Polymerase Chain Reaction (RT–PCR)

A 100 mg sample was taken, total RNA was extracted from the tissue according to the manufacturer's instructions, amplified by a heat denaturation-retroactivity-extension cycle [7], and PCR products were electrophoresed on a 1.5% agarose gel and visualized using a gel imaging system. The relative expression of each mRNA was calculated using the 2^−ΔΔCt^ relative quantification method.

### 2.6 Statistical Analysis

Data were analyzed by one-way ANOVA(one-way analysis of variance). Relative values of mNSS score, alternate correct rate, protein and mRNA were expressed as mean ± SD for each group of rats. Differences between groups were determined by LSD or Dunnett's test. SPSS software (version 22.0, Chicago, IL, USA) was used for statistical analysis of the data. p-value < 0.05 was considered a statistically significant difference.

## Ethics Statement

The animal study was reviewed and approved by the Medical Ethics Committee of First Hospital of Qinhuangdao in China (ID Number: 20,140,018). All animal experimental procedures complied with the ARRIVE guidelines and were carried out in accordance with the U.K. Animals (Scientific Procedures) Act, 1986 and associated guidelines.

## CRediT authorship contribution statement

**Xinyu Yao:** Conceptualization, Methodology, Software, Data curation, Writing – original draft. **Wenzhu Wang:** Methodology, Resources, Investigation. **Yue Li:** Conceptualization, Methodology, Software, Data curation, Writing – original draft. **Zhendong Cao:** . **Yongheng Wang:** Conceptualization, Validation, Formal analysis. **Yi Yuan:** Writing – review & editing, Formal analysis. **Xiaoling Li:** Writing – review & editing, Formal analysis. **Xin Liang:** Conceptualization, Validation, Formal analysis. **Lanxiang Liu:** Funding acquisition, Supervision, Project administration. **Yan Yu:** Methodology, Resources, Investigation.

## Declaration of Competing Interest

The authors declare that they have no known competing financial interests or personal relationships that could have appeared to influence the work reported in this paper.

## Data Availability

Data of MSCs combined with LITUS treatment to improve cognitive impairment in a moderate traumatic brain injury model in rats (Original data) (Mendeley Data).
